# Cross-sectional survey of parental barriers to participation in pediatric participant research registries

**DOI:** 10.1371/journal.pone.0268553

**Published:** 2022-05-18

**Authors:** Rebecca A. Slotkowski, Shirley F. Delair, Kari A. Neemann

**Affiliations:** 1 College of Medicine, University of Nebraska Medical Center, Omaha, Nebraska, United States of America; 2 Department of Pediatrics, Division of Infectious Disease, University of Nebraska Medical Center, Omaha, Nebraska, United States of America; Flinders University, AUSTRALIA

## Abstract

Research registries are a powerful tool for boosting recruitment into clinical trials. However, little is known about how parents approach the decision to enroll their child in a pediatric participant research registry (PPRR). We conducted in-person, written, or telephone surveys with parents/guardians of children hospitalized at Children’s Hospital of Omaha, Nebraska to identify attitudes towards and barriers to enrollment in PPRRs. Overall, our population (N = 36) had positive attitudes toward PPRRs, with 77.8% (CI: 61.6, 88.4) of participants stating they were “somewhat” or “very” likely to enroll their child. Likelihood to enroll differed between various recruitment and enrollment methods, with participants stating they would be more likely to enroll their child in a PPRR if they were recruited by their child’s primary care provider or a nurse in clinic (p = 0.02) and less likely to enroll if they were recruited through social media (p<0.001). Additionally, over 90% of participants who were likely to enroll their child in a PPRR (N = 28) were also willing to provide demographic, medical, and lifestyle information. However, these participants remained concerned about inappropriate sharing of their information with insurance or for-profit companies (53.6%, CI: 35.8, 70.4) and about receiving unwanted telephone calls from the registry (78.6%, CI: 60.0, 90.0). Parents are generally willing to enroll their child in a PPRR. However, to optimize enrollment, investigators must understand parental preferences for and concerns surrounding enrollment in a PPRR.

## Introduction

Participant recruitment into clinical trials can be a challenging endeavor. An estimated 22% of all phase III clinical trials sponsored by the National Cancer Institute from 2000 to 2007 failed to meet accrual goals due to insufficient accrual rates [1]. Similarly, 19% percent of phase II and III trials registered as closed in the Canadian National Library of Medicine by 2011 were either terminated due to insufficient accrual or finished with <85% anticipated enrollment [2] and approximately 25% of randomized clinical trials in Switzerland were prematurely discontinued, with insufficient accrual most often cited as the reason for trial termination [3]. Many reasons for this high rate of recruitment failure have been suggested, including inadequate advertising strategies to identify eligible individuals and lack of access to individuals who are both eligible for and interested in participation [4].

In recent years, participant research registries have emerged as an effective tool to streamline the recruitment process and avoid some common reasons for recruitment failure. Research registries provide researchers with a ready-made list of individuals who meet basic eligibility requirements and have indicated a potential interest in research participation [5–10]. Multiple models of research registries exist, each with different focuses for recruitment. National registries provide researchers access to an especially large pool of individuals who have indicated an interest in research [5]. Regional registries can be useful for recruiting a cohort that matches the demographic profile of the region, particularly in regions with a high proportion of individuals who are notoriously underrepresented in research such as racial and ethnic minorities or rural communities [6]. Disease-specific registries may be more useful in recruiting participants with rare diseases [7, 8] or in pre-screening participants for specific eligibility criteria [9].

Although research registries are rapidly becoming an invaluable recruitment tool to aid clinical trials in meeting their accrual goals, registries are themselves limited by their ability to recruit participants. Successful strategies to enroll participants into research registries include recruitment by health care personnel during a scheduled medical appointment [9], prompts through an electronic health record [6], community-based in-person recruitment events [6], and social media [8]. The relative success of a chosen recruitment strategy can vary greatly between populations based on the specific barriers to enrollment that population faces. For example, 30% of individuals who enrolled in a state-wide research registry for Arkansas residents reported that they first heard about the registry from an electronic medical record prompt or community outreach at a state fair while only 2% reported that they first heard about the registry through social media [6]. In contrast, 48% of individuals who enrolled in a registry for patients with neurofibromatosis type I reported that they first heard about the registry from Facebook while only 12% reported that they first heard about the registry through health care personnel or a community-based advocacy group [8]. This discrepancy highlights the need for further research into population-specific preferences for enrollment in research registries [11].

To our knowledge, no previous research has been published on parental barriers to enrollment in pediatric participant research registries (PPRRs). The aim of this study was to explore parental attitudes towards and barriers to PPRR recruitment, enrollment, and communication practices in order to inform the design of or maximize participation in PPRRs.

## Methods

This cross-sectional study was approved with a waiver for written consent by the University of Nebraska Medical Center Institutional Review Board. Verbal consent to participate in this research was obtained prior to administering the survey. The Checklist for Reporting of Survey Studies (CROSS) was utilized to ensure the results of this study were reported accurately and ethically [12].

### Participant eligibility

Participants were included in the survey if they were at least 19 years of age and were parents or guardians of children hospitalized at Children’s Hospital & Medical Center in Omaha, Nebraska during the study period (July through August 2020). To respect the privacy of families with very ill children, parents of children hospitalized in an intensive care unit were excluded from this study.

### Survey procedures

During the study period, eligible participants were approached in person and asked if they would like to take a written survey or complete a telephone or in-person interview. Surveys were administered by the method requested by the parent/guardian. A paper copy of the survey was left with parents who wished to complete the written survey independently and completed surveys were collected the next day. Throughout the survey period, telephone and in-person surveys were conducted by a single, trained research staff member within one day of the parent/guardian agreeing to participate in the study. Surveys were completed over the telephone for participants requiring interpreter services, secondary to the need to conserve PPE during the COVID-19 pandemic. Study data were collected and managed using the REDCap electronic data capture tools hosted at the University of Nebraska Medical Center to protect survey anonymity and confidentiality. Service and support is provided by the Research Information Technology Office (RITO), which is funded by the Vice Chancellor for Research. Data was independently reviewed by research staff to minimize risk of human error in data entry.

### Survey measures

Survey questions were adapted from Grill et al. [11] and available in S1 Questionnaire. To assess participant likelihood to enroll in a PPRR, participants were asked how likely they were to enroll in a PPRR. This was considered the baseline likelihood of enrollment. Participants were then asked how likely they would be to enroll their child in a PPRR if health information was provided as an incentive for enrollment, a specific recruitment method was utilized (social media, nurse in clinic, telehealth nurse, or primary care provider), or a specific enrollment method was the only option for enrollment into the registry (phone, website, website requiring a username and password, or email). Participant likelihood to enroll for each recruitment or enrollment method was compared to baseline likelihood of enrollment.

The surveys also assessed demographic characteristics (race/ethnicity, language spoken at home, education, employment status, marital status, zip code, income, and prior research experience), preferred enrollment logistics (maximum time for enrollment, acceptable information to collect, maximum contact frequency, and acceptable/preferred methods of communication), concerns surrounding inappropriate sharing of information (with personnel, insurance companies, and for profit companies), and concerns surrounding unwanted contact from the registry (via phone, email, and letter). The demographic characteristics and preferred enrollment logistic questions used a multiple-choice format, while all other survey questions used a 4-point rating scale with (1) “very unlikely”, (2) “somewhat unlikely”, (3) “somewhat likely”, and (4) “very likely”. Participants were considered likely to enroll their child in a PPRR if they replied (3) “somewhat likely” or (4) “very likely”.

### Statistical analysis

Sample proportions were reported with Modified Wald Confidence Intervals. Mann-Whitney U tests were used to evaluate statistical differences for all survey responses, excluding questions about acceptable and preferred method of communication, across demographic variables. Chi square goodness of fit tests were used to compare baseline participant likelihood to enroll versus likelihood to enroll using different recruitment or enrollment methods and likelihood to enroll if pediatric health research updates were provided as an incentive for enrollment. Chi square tests for independence were used to determine if significant differences in acceptable and preferred methods of communication with registry staff existed across demographic variables. Significant results were defined as p<0.05 for the chi square tests of independence and chi square goodness of fit test or U values less than the critical value at α = 0.05 for the Mann-Whitney U tests. All statistical calculations were performed using Excel software.

Participants who declined to provide their net monthly income (N = 6) were excluded from income-related analyses but included in all other statistical tests. If participants provided a range for estimated net monthly salary, the lowest estimate was used for statistical analysis. Participants were separated into “at or below the poverty line” and “above the poverty line” based on the 2020 US Federal Poverty Guidelines [13]. For analysis by zip code, respondents were separated into rural or urban groups based on Federal Office of Rural Health Policy guidelines [14]. For analysis by race/ethnicity, participants were divided into a non-Hispanic (NH) White and a non-White group, where non-White included respondents that identified as Hispanic ethnicity. If two parents or guardians were available to answer the questionnaire, demographic information from the parent with the higher level of education was used.

## Results

### Demographic characteristics

Of the 57 parents or guardians approached, 36 completed the survey. The majority of our sample population were employed, married, NH-White individuals from urban communities and without any prior experience participating in research (Table 1). Participant age ranged from 21 to 62 years old, with a median age of 37.

**Table 1:** Demographic characteristics.

Characteristic	N	%
**Race/Ethnicity**		
NH-White	25	69.4
Hispanic	5	13.9
African American	3	8.3
Native American	1	2.8
Asian/Pacific Islander	2	5.6
**Language Spoken at Home**		
English	32	88.9
Spanish	2	5.6
Other	2	5.6
**Highest Level of Education**		
High School or Less	7	19.4
Completed Some College	8	22.2
Associates Degree	5	13.9
Bachelor’s Degree	7	19.4
Advanced Degree	9	25.0
**Marital Status**		
Married or in Domestic Partnership	24	66.7
Divorced or Separated	3	8.3
Single	9	25.0
**Employment Status**		
Employed Full-Time	18	50.0
Employed Part-Time	3	8.3
Self-Employed	2	5.6
Homemaker	9	25.0
Unemployed, Disabled, or Retired	4	11.1
**Geographic Location**		
Rural	10	32.1
Urban	26	67.9
**Prior Research Experience**		
Familial Research Experience	8	22.2
No Prior Experience	28	77.8
**Income**		
At/Below Poverty Line	11	30.6
Above Poverty Line	19	52.8
Declined to Provide	6	16.7

### Participant likelihood to enroll their child in a PPRR

**Fig 1:**
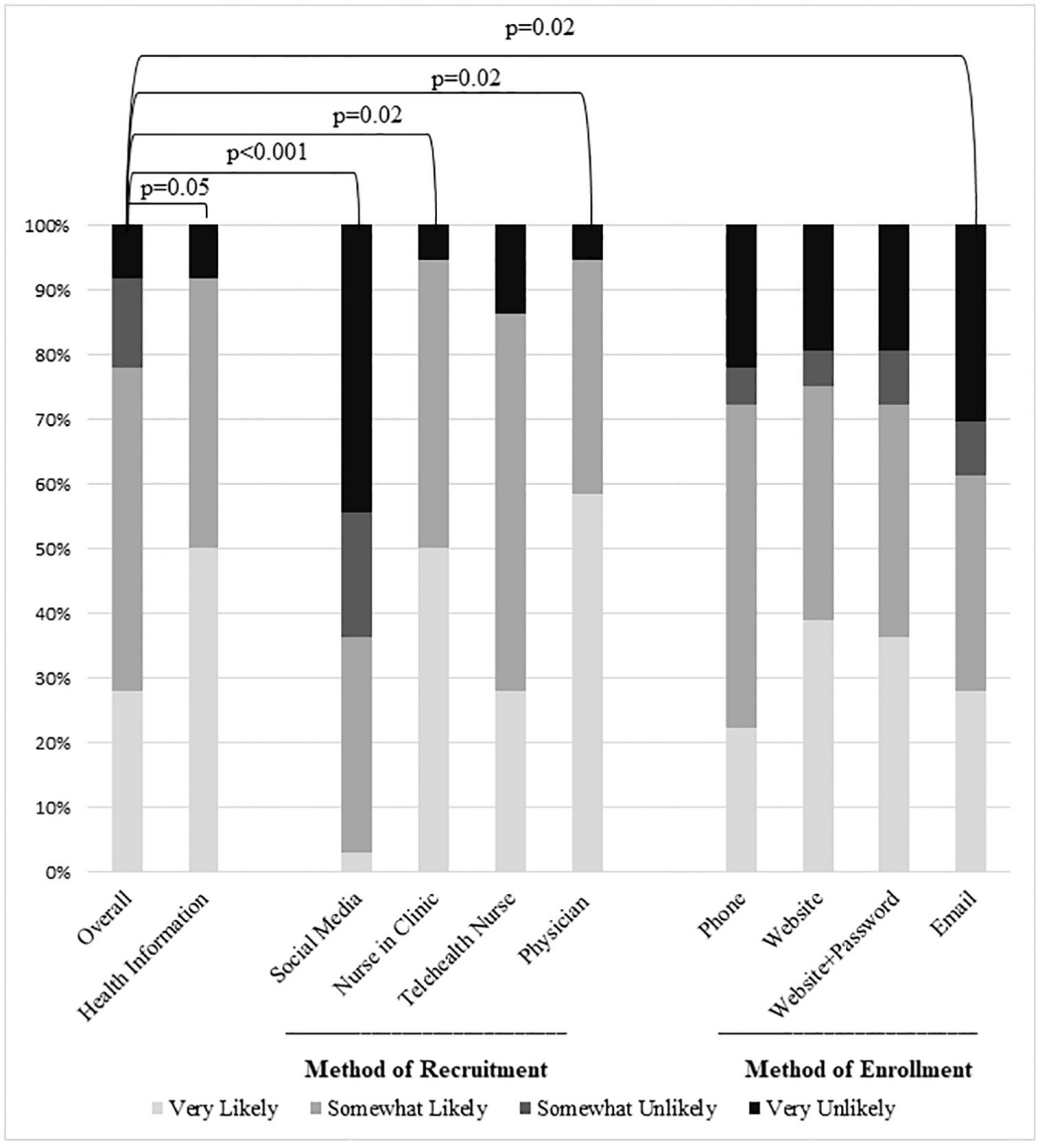
Differences in participant likelihood to enroll their child in a PPRR based on whether they received information on pediatric health as a benefit of enrollment, method of recruitment, and method of enrollment (N = 36).

Of the 36 parents/guardians surveyed, 28 stated that they would be likely to enroll their child in a PPRR (77.8%, CI: 61.6, 88.4) (Fig 1). This was considered the baseline likelihood to enroll. No significant differences in baseline participant likelihood to enroll were noted for any assessed demographic variable.

If pediatric health information updates based on current medical research were offered as an incentive to enroll in a PPRR, 33 out of the 36 survey respondents were likely to enroll their child (91.7%, CI: 77.3, 97.7, p = 0.05) (Fig 1). However, participants with household incomes at or below the poverty line were less likely to enroll compared to participants with household incomes above the poverty line if health updates were offered as an incentive to enroll (8 of 11 vs 19 of 19, p = 0.04). There were no other significant differences noted for the assessed demographic variables.

### Effect of recruitment method on participant likelihood to enroll their child in a PPRR

If recruitment was offered by the child’s primary care provider or a nurse during a physical clinic appointment, 34 out of the 36 participants were likely to enroll their child in a PPRR (94.4%, CI: 80.7, 99.3, p = 0.02). In contrast, only 13 participants were likely to enroll their child in a PPRR if they saw the registry advertised on social media (36.1%, CI: 22.5, 52.5, p<0.001). Compared to the baseline likelihood of enrollment, there was no significant difference in likelihood to enroll if participants were recruited by a telehealth nurse (86.1%, CI: 707.7, 94.3, p = 0.2) (Fig 1). Respondents with incomes at or below the poverty line were less likely to enroll their child in a PPRR than respondents above the poverty line when registration was offered by their child’s primary care provider or a nurse during a physical clinic appointment (9 of 11 vs 19 of 19, p = 0.04). There were no other significant differences noted for the assessed demographic variables.

### Effect of enrollment method on participant likelihood to enroll their child in a PPRR

Compared to the baseline likelihood of enrollment, participants were less likely to enroll their child in a PPRR if enrollment was only offered through email (22 of 36, 61.1%, CI: 44.8, 75.2, p = 0.02). There was no significant difference in participant likelihood to enroll if enrollment was offered through telephone (26 of 36, 70.2%, CI: 55.8, 84.2, p = 0.4), a website (27 of 36, 75%, CI: 58.7, 86.3, p = 0.7), or a website requiring a username and password (26 of 36, 70.2%, CI: 55.8, 84.2, p = 0.4) (Fig 1). Non-White participants were significantly less likely to enroll their child in a PPRR compared to NH-White participants if enrollment was only offered via a website (5 of 11 vs 22 of 25, p = 0.03), a website requiring a username and password (5 of 11 vs 21 of 25, p = 0.05), or email (4 of 11 vs 18 of 25, p = 0.01). Participants with household incomes at or below the poverty line were significantly less likely to enroll their child in a PPRR compared to participants with incomes above the poverty line if enrollment was only offered via a website (6 of 11 vs 16 of 19, p = 0.04) or email (4 of 11 vs 16 of 19, p = 0.01). There were no other significant differences noted for the assessed demographic variables.

### Maximum enrollment time among participants who are likely to enroll their child in a PPRR

Survey participants who reported that they were likely to enroll their child in a PPRR (N = 28) were questioned about their preferred logistics for enrollment. Most participants (23 of 28, 82.1%, CI: 63.8, 92.4), stated that they would be willing to spend a maximum of 5–30 minutes enrolling their child (Table 2). There were no significant differences noted for maximum enrollment time across the assessed demographic variables.

**Table 2:** Parental preferences for enrollment logistics and communication.

Characteristic	N	%	CI
**Maximum Acceptable Time to Enroll**			
5 Minutes	5	17.9	7.6–36.2
15 Minutes	10	35.7	20.7–54.3
30 Minutes	8	28.6	15.2–47.3
60 Minutes	2	7.1	1.0–24.0
90 Minutes	3	10.7	3.0–28.2
**Acceptable Information to Collect**			
Demographic	26	92.9	76.0–99.0
Lifestyle	27	96.4	80.5–100
Self-Reported Medical	26	92.9	76.0–99.0
Medical Records	25	89.3	71.8–97.0
**Acceptable Contact Schedule**			
Never	1	3.6	0–19.47
Annual	10	35.7	20.7–54.3
Every 4–6 Months	6	21.4	10.0–40.0
Monthly	2	7.1	1.0–24.0
No Limit	9	32.1	17.9–50.8
**Acceptable Method of Communication**			
None	1	3.6	0–19.5
Phone Call	25	89.3	71.8–97.0
Text	21	75.0	56.3–87.5
Email	21	75.0	56.3–87.5
Letter	18	64.3	45.7–79.3
**Preferred Method of Communication**			
None	1	3.6	0–19.5
Phone Call	7	25.0	12.5–43.7
Text	6	21.4	10.0–40.0
Email	7	25.0	12.5–43.7
Letter	8	28.6	15.2–47.3

### Willingness to provide personal information among participants who are likely to enroll their child in a PPRR

Nearly all participants who were likely to enroll their child in a PPRR were willing to provide lifestyle (27 of 28, 96.4%, CI: 80.5, 100), demographic (26 of 28, 92.9%, CI: 76.0, 99.0), and self-reported medical (26 of 28, 92.9%, CI: 76.0, 99.0) information. Additionally, 25 of the 28 participants who were likely to enroll their child in a PPRR (89.3%, CI: 71.8, 87.0) were also willing to allow researchers to link their child’s medical records to a PPRR (Table 2). Non-White participants were significantly less willing to provide demographic (7 of 9 vs 19 of 19, p = 0.05) and self-reported medical (7 of 9 vs 19 of 19, p = 0.01) information about their child compared to NH-White participants. There were no other significant differences noted for willingness to provide personal information across the assessed demographic variables.

### Acceptable contact schedule among participants who are likely to enroll their child in a PPRR

Participants who were likely to enroll their child in a PPRR were most often willing to be contacted by the registry annually (10 of 28, 35.7%, CI: 20.7, 54.3). However, a similar number of participants (9 of 28, 32.1%, CI: 19.9, 50.8) reported that they would like to be contacted by the registry whenever a study applicable to their child emerged, without limitations based on contact schedule. One participant (3.6%, CI: 0, 19.5) stated they would never want to be contacted by the registry, despite having reported that they were likely to enroll their child in a PPRR (Table 2). There were no significant differences noted for acceptable contact schedules across the assessed demographic variables.

### Acceptable and preferred communication methods among participants who are likely to enroll their child in a PPRR

A telephone call was most often reported as an acceptable method of communication (25 of 28, 89.3%, CI: 71.8, 97.0). However, 21 participants (75.0%, CI: 56.3, 87.5) also reported text or email as an acceptable method of communication and 18 participants (64.3%, CI: 45.7, 79.3) reported a mailed letter as acceptable (Table 2). Several demographic variables influenced the acceptability of the different communication methods. Participants with household incomes at or below the poverty line were less likely to report that text or email were acceptable methods of communication (3 of 7 vs 15 of 17, p = 0.02) and participants who were not employed were less likely to state that email was an acceptable method of communication (4 of 10 vs 17 of 18, p = 0.001). A letter was less likely to be considered an acceptable method of communication for non-White participants (3 of 9 vs 15 of 19, p = 0.02) as well as participants with less than a college degree (4 of 12 vs 14 of 16, p = 0.003) or no prior research experience (11 of 21 vs 7 of 7, p = 0.02).

Participants were then asked to choose their preferred method of communication from their listed acceptable methods of communication. The preferred method of communication with PPRR staff was split nearly equally between a mailed letter (8 of 28, 28.6%, CI: 15.2, 47.3), telephone call (7 of 28, 25.0%, CI: 12.5, 43.7), text message (6 of 28, 21.4%, CI: 10.0, 40.0), and email (7 of 28, 25.0%, CI: 12.5, 43.7) (Table 2). Participants younger than the median sample age (<37) were less likely to report a letter was their preferred method of contact compared to participants who were older than the median age (1 of 13 vs 7 of 15, p = 0.02). There were no other significant differences noted for preferred or acceptable contact methods across the assessed demographic variables.

### Privacy concerns among participants who are likely to enroll their child in a PPRR

Of the 28 participants who were likely to enroll their child in a PPRR, 12 (42.8%, CI: 26.6, 60.9) reported concerns that their information would be shared inappropriately with other in-network health care personnel and 15 (53.6%, CI: 35.8, 70.4) reported concerns that their information would be shared with out-of-network health care personnel, insurance companies, or for-profit companies. Participants who were likely to enroll their child in a PPRR were as likely to report concerns about inappropriate sharing of information as participants who were unlikely to enroll their child in a PPRR (p>0.2) (Table 3). Participants were more likely to report concerns about inappropriate sharing of information with out-of-network health care personnel if they were non-White (p = 0.01), had no prior familial research experience (p = 0.002), were unemployed (p = 0.01), or lived in a rural area (p = 0.05) (S1 Table). When it comes to inappropriate sharing of information with for-profit companies, participants who were non-White (p = 0.02), had no prior familial research experience (p = 0.04), or were unemployed (p = 0.01) were more likely to report concerns (S1 Table). Unemployed participants were also more likely to report concerns about inappropriate sharing with in-network health care personnel (p = 0.03) or insurance companies (p = 0.002) (S1 Table). There were no other significant differences noted for parental privacy concerns across the assessed demographic variables.

**Table 3:** Parental privacy concerns.

	Likely to Enroll (N = 28)			Unlikely to Enroll (N = 8)			
Sharing Concern	N	%	CI	N	%	CI	P
In-Network Personnel	12	42.9	26.6–60.9	2	25.0	6.7–60.0	>0.2
Out-of-Network Personnel	15	53.6	35.8–70.4	2	25.0	6.7–60.0	>0.2
Insurance Companies	15	53.6	35.8–70.4	5	62.5	30.4–86.2	>0.2
For-Profit Companies	15	53.6	35.8–70.4	5	62.5	30.4–86.2	>0.2

### Concerns surrounding unwanted communication among participants who are likely to enroll their child in a PPRR

**Fig 2:**
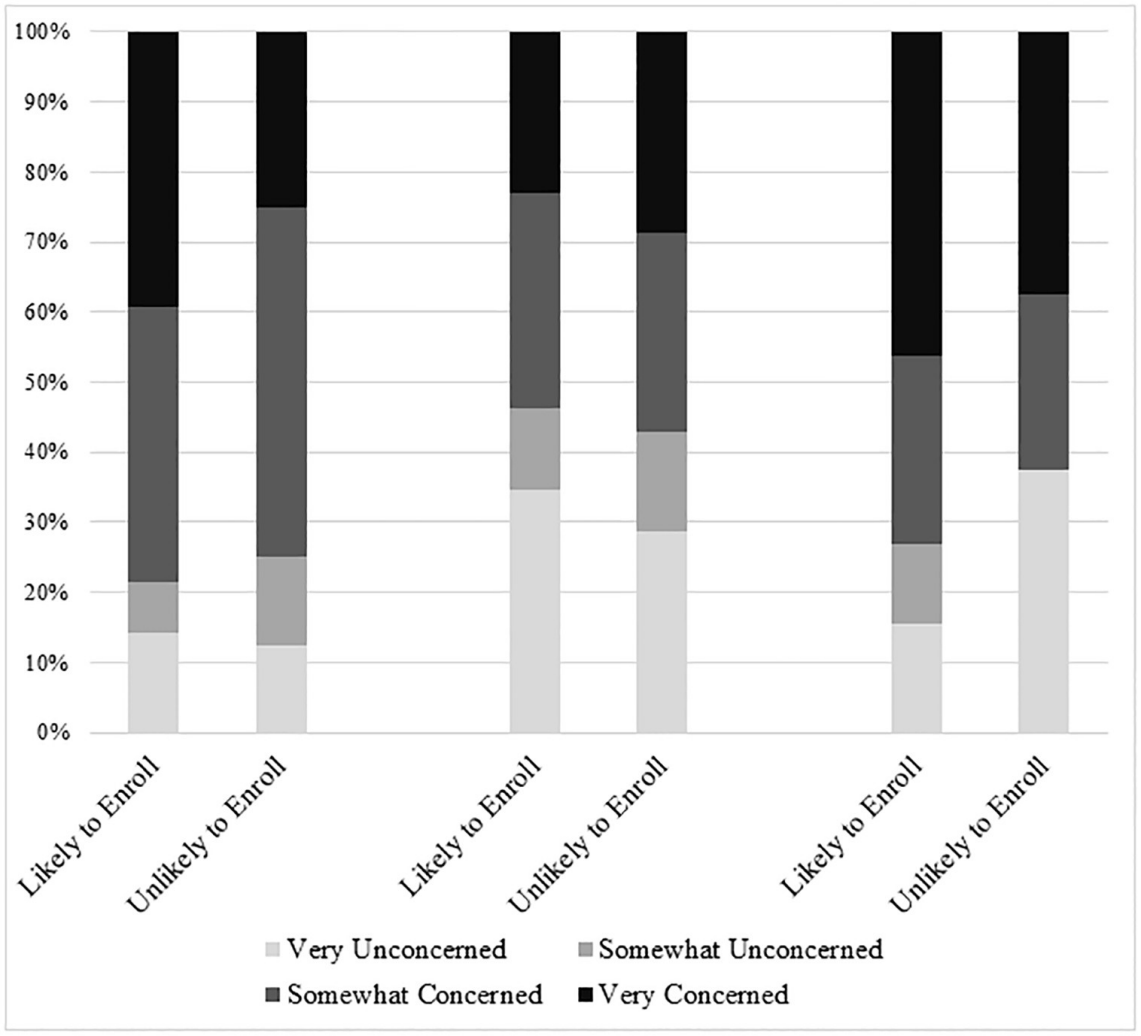
Concerns surrounding unwanted communication among survey participants.

Of the 28 participants who were likely to enroll their child in a PPRR, 22 (78.6%, CI: 60.0, 90.0) were concerned that they would receive unwanted telephone calls after enrollment, 21 (75.0%, CI: 56.3, 87.5) were concerned that they would receive unwanted emails, and 16 (57.1%, CI: 39.1, 73.4) were concerned that they would receive unwanted letters. Concerns were similar between participants who were likely to enroll their child in a PPRR compared to participants who were unlikely to enroll for all methods of communication (p>0.2) and spanned all demographic backgrounds, with no significant differences in any of the demographic variables assessed (Fig 2).

## Discussion

Most survey participants were at least “somewhat” likely to enroll their child in a PPRR, especially if parents received pediatric health information updates based on current medical research as an incentive for enrollment. Consistent with previous studies of parent preferences for recruitment into singular pediatric clinical trials [15–17], parents were more likely to enroll their child in a PPRR if enrollment was offered by the child’s primary care provider or a nurse during an in-person medical appointment. However, recruitment during medical appointments can be prohibitively time-intensive for staff [15, 18]. Advertisement on social media has been suggested as an alternative method to quickly and inexpensively offer enrollment in a PPRR to a large number of families [8]; however, the majority of parents/guardians in our survey were unlikely to enroll their child in a PPRR which they saw advertised on social media.

Other proposed recruitment strategies focus on incentivizing physicians to participate in research recruitment and providing more institutional support for physicians who are interested in research [18]. We suggest the following additional strategies to appropriately balance available physician resources with parental preferences in PPRR recruitment methods: (a) increasing physician “buy-in” [18] by recruiting primary care physicians as sub-investigators in the development of PPRRs; (b) decreasing the economic burden of devoting clinic time to research recruitment [18] by advocating for “discussion of potential study” as a billable endeavor under Federal guidelines; or (c) minimizing physician time requirements [15, 18] by implementing the use of a screening question added on to existing new-patient paperwork which will allow parents to opt-in to receive further information about PPRRs from PPRR staff.

Although recruitment into a PPRR by a primary care provider appeared to be a particularly effective recruitment strategy for the majority of participants in our study, non-White participants were significantly less likely to enroll their child in a PPRR compared to NH-White participants if enrollment was offered by their child’s primary care provider. Additionally, non-White participants and participants with incomes at or below the federal poverty guidelines were significantly less likely to enroll their child in a PPRR if enrollment was only offered through electronic methods and participants with incomes at or below the federal poverty guidelines were significantly less likely to report that email or text were acceptable modalities of communication with a PPRR. These findings emphasize the importance of developing recruitment and enrollment strategies that address accessibility concerns [19]. Strategies to improve the enrollment of racial/ethnic minorities [20–22], individuals with a low-income, and other marginalized populations with historically poor access to health care [23] should include: (a) acknowledging that these at-risk communities have a high prevalence of adverse health care experiences [23, 24]; (b) providing training for PPRR staff in unconscious bias and culturally-appropriate communication [20]; (c) ensuring the PPRR staff come from diverse racial ethnic backgrounds and lived experiences to increase understanding of the community and trust [19]; (d) work in conjunction with trusted community leaders to promote trust and collaboration between the PPRR and the community [19]; and (e) offering multiple modalities for enrollment to address barriers such as lack of transportation (for in-person registration) [19] or lack of access to the internet (for electronic registration) [25].

In addition to designing appropriate recruitment and enrollment strategies, PPRRs must address common parental barriers to enrollment in a PPRR including fear of receiving unwanted communications and privacy concerns. In our population, over 50% of participants who were likely to enroll their child in a PPRR were concerned that their information would be inappropriately shared with out-of-network health care personnel, insurance companies, or for-profit companies. This is consistent with previous studies, where 41% [26] to 70% [16] of parents reported privacy concerns as a barrier to enrollment in clinical research. Unwanted communication was even more concerning for our population. Almost 80% of participants who were likely to enroll their child in a PPRR were concerned that they would receive unwanted phone calls after enrollment. A previous study revealed that access to a preference-setting tool increased parental satisfaction with the process of participation in a singular pediatric clinical trial [27], and we suggest that allowing participants to customize their communication and privacy settings during enrollment in a PPRR may mitigate concerns about unwanted communication and inappropriate sharing of information. Additionally, we suggest that PPRRs should have a mechanism to record when families are contacted by a study investigator to avoid overwhelming families or greatly exceeding their preferred contact frequency.

There are several limitations to this study that should be addressed. This study had a small sample size, which may limit statistical power. Generalizability may be reduced as it was conducted in an inpatient population at a single medical center. However, the racial/ethnic demographic in the study was similar to that of the state of Nebraska [28]. Additionally, participants were given an option to complete a written, telephone, or in-person survey in order to promote accessibility to participation in this study. However, there is a possibility that response bias varied between the written, telephone, and in-person survey methods. There is also the possibility that a non-response bias exists, although we achieved a relatively high response rate of 63%.

The results of this study will guide the development of future PPRRs. Our survey results show that parents are willing to enroll their child in a PPRR if appropriate strategies are implemented to ensure enrollment is convenient for families and limits communication and privacy concerns. To optimize involvement, PPRRs must have culturally sensitive recruitment practices, flexible enrollment procedures, and a mechanism to solicit and respect individual parental preferences for communication and privacy practices. Future studies should investigate if our survey of parents receiving inpatient medical care for their child in Omaha, Nebraska accurately depict the nuances of parental preference for enrollment in PPRRs for other regions of the United States and structured interventional studies should be assessed to determine if implementing these strategies to improve compliance with parental preferences leads to higher enrollment in PPRRs.

## Supporting information

S1 Questionnaire Potential barriers to participant registry questionnaire. (DOCX)

S1 Table Demographic differences in parental privacy concerns among parents who are likely to enroll their child in a PPRR. (DOCX)

S1 Data (XLSX)
