# Supplementary material for: Cross-sectional survey of parental barriers to participation in pediatric participant research registries
Source: PLoS One. 2022 May 18;17(5):e0268553. doi: 10.1371/journal.pone.0268553 (PMC9116665; doi:10.1371/journal.pone.0268553)
Supplement: S1 Table — (DOCX) [file pone.0268553.s002.docx]

|  | **Likely to Enroll and**  **Non-White (N=9)** | | | | **Likely to Enroll and NH-White (N=19)** | | | |  |
| --- | --- | --- | --- | --- | --- | --- | --- | --- | --- |
| **Sharing Concern** | **N** | **%** | **CI** | **N** | | **%** | **CI** | **P** | |
| In-Network Personnel | 6 | 66.7 | 35.1-88.0 | 6 | | 31.6 | 15.3-54.2 | >0.2 | |
| Out-of-Network Personnel | 8 | 88.9 | 54.0-99.8 | 7 | | 36.8 | 19.2-59.1 | 0.01 | |
| Insurance Companies | 6 | 66.7 | 35.1-88.0 | 9 | | 47.4 | 27.4-68.2 | 0.08 | |
| For-Profit Companies | 7 | 77.8 | 44.1-94.3 | 8 | | 42.1 | 23.2-63.7 | 0.02 | |
|  | **Likely to Enroll and Rural (N=9)** | | | | **Likely to Enroll and Urban** (**N=19)** | | | |  |
| **Sharing Concern** | **N** | **%** | **CI** | **N** | | **%** | **CI** | **P** | |
| In-Network Personnel | 5 | 55.6 | 26.7-80.9 | 7 | | 36.8 | 19.2-59.1 | >0.2 | |
| Out-of-Network Personnel | 7 | 77.8 | 44.1-94.3 | 8 | | 42.1 | 23.2-63.7 | 0.05 | |
| Insurance Companies | 6 | 66.7 | 35.1-88.0 | 9 | | 47.4 | 27.4-68.2 | >0.2 | |
| For-Profit Companies | 7 | 77.8 | 44.1-94.3 | 8 | | 42.1 | 23.2-63.7 | 0.1 | |
|  | **Likely to Enroll and Not Employed (N=10)** | | | | **Likely to Enroll and Employed (N=18)** | | | |  |
| **Sharing Concern** | **N** | **%** | **CI** | **N** | | **%** | **CI** | **P** | |
| In-Network Personnel | 7 | 70.0 | 39.2-89.4 | 5 | | 27.8 | 12.4-51.3 | .03 | |
| Out-of-Network Personnel | 9 | 90.0 | 57.1-100 | 6 | | 33.3 | 16.3-56.5 | .01 | |
| Insurance Companies | 9 | 90.0 | 57.1-100 | 6 | | 33.3 | 16.3-56.5 | 0.002 | |
| For-Profit Companies | 9 | 90.0 | 57.1-100 | 6 | | 33.3 | 16.3-56.5 | 0.005 | |
|  | **Likely to Enroll With Research Experience (N=7)** | | | | **Likely to Enroll Without Research Experience (N=21)** | | | |  |
| **Sharing Concern** | **N** | **%** | **CI** | **N** | | **%** | **CI** | **P** | |
| In-Network Personnel | 1 | 14.3 | 1.0-53.6 | 11 | | 52.4 | 32.4-71.6 | 0.1 | |
| Out-of-Network Personnel | 1 | 14.3 | 1.0-53.6 | 14 | | 66.7 | 45.2-82.8 | 0.002 | |
| Insurance Companies | 3 | 42.9 | 16.0-74.9 | 12 | | 57.1 | 36.5-75.5 | >0.2 | |
| For-Profit Companies | 2 | 28.6 | 7.9-64.8 | 13 | | 61.9 | 40.8-79.2 | 0.04 | |
