# Supplementary material for: Cross-sectional survey of parental barriers to participation in pediatric participant research registries
Source: PLoS One. 2022 May 18;17(5):e0268553. doi: 10.1371/journal.pone.0268553 (PMC9116665; doi:10.1371/journal.pone.0268553)
Supplement: Supplementary file 2 [file pone.0268553.s001.docx]

Parental Barriers to Participant Registry

Record ID ______________________________

As part of the Nebraska Pediatric Clinical Trials Unit, the Nebraska Pediatric Research Registry was created to collect and maintain information from Nebraska families who may want to participate in research in the future. Families are then contacted when an opportunity to participate in a clinical study that is relevant to their child becomes available. Participating in a registry does not automatically enroll your child in a study; it's just allows you to be aware of the opportunities available.

Today, we are asking for your assistance and input, to help us learn about how people approach the decision of whether to enroll in a research registry. We are inviting you to participate in our survey. This survey is considered research. All responses are confidential and only compiled results will be reported. You will not need to enroll in the Nebraska Pediatric Research Registry once it is complete, but you are welcome to do so if desired.

IRB# 470-20-EX

Age of Parent/Guardian (years) ______________________________

Race (mark all that apply)
- ☐ White
- ☐ Hispanic/Latino
- ☐ Black/African American
- ☐ Native American/American Indian
- ☐ Asian/Pacific Islander
- ☐ Other

Race (Other) ______________________________

Ethnicity
- ☐ Hispanic or Latino or Spanish Origin
- ☐ Not Hispanic or Latino or Spanish Origin

What is the primary language spoken in your home?
- ◯ English
- ◯ Spanish
- ◯ French
- ◯ Arabic
- ◯ Korean
- ◯ Chinese
- ◯ Other

Language (Other) ______________________________

What is your marital status?
- ◯ Single (never married)
- ◯ Married or in a domestic partnership
- ◯ Separated
- ◯ Divorced
- ◯ Widowed

How many children are in the household? ______________________________

What is your highest level of education?	◯ Did not attend school<br>◯ Attended some school<br>◯ Graduated High School<br>◯ Completed some college<br>◯ Trade/technical/vocational training<br>◯ Associate's degree<br>◯ Bachelor's degree<br>◯ Master's degree<br>◯ Professional degree<br>◯ Doctorate degree
What is your current employment status?	◯ Employed Full Time<br>◯ Employed Part Time<br>◯ Unemployed<br>◯ Student<br>◯ Retired<br>◯ Homemaker<br>◯ Self-employed<br>◯ Unable to work secondary to disability<br>◯ Unable to work without disability
What is your approximate monthly take-home pay?	______________________________
Which Zip Code does your child primarily reside?	______________________________
Is your child enrolled in a health insurance plan?	◯ Yes<br>◯ No
What is your primary mode of transportation?	◯ Your own private vehicle<br>◯ A friend/family member private vehicle<br>◯ Public transportation (bus, cab, Uber, etc)
Do you have a smart phone?	◯ Yes<br>◯ No
Do you have difficulty maintaining your monthly phone access?	◯ Yes<br>◯ No
On average, how many times a year does your child visit their primary physician's office?	______________________________
Would you agree that vaccines protect children from potentially life-threatening disease?	◯ Yes<br>◯ No
Has your child ever participated in health-based research?	◯ Yes<br>◯ No
Was it a positive experience?	◯ Yes<br>◯ No
Have your or another family member ever participated in health-based research?	◯ Yes<br>◯ No
Was it a positive experience?	◯ Yes<br>◯ No

Would the current COVID-19 pandemic influence your decisions on whether or not to participate in research?

◯ Yes
◯ No

Please select the answer that best represents your feelings and opinions. Unless otherwise stated, please select only one answer for each of the following questions.

How likely would you be to enroll your child in a potential participants registry?

◯ Very Unlikely
◯ Somewhat Unlikely
◯ Somewhat Likely
◯ Very Likely

How likely would you be to enroll your child in a registry if it was only offered through the telephone (if you had to call a number and answer questions)?

◯ Very Unlikely
◯ Somewhat Unlikely
◯ Somewhat Likely
◯ Very Likely

How likely would you be to enroll your child in a registry if it was only offered through the Internet (if you had to go to a website and answer questions)?

◯ Very Unlikely
◯ Somewhat Unlikely
◯ Somewhat Likely
◯ Very Likely

How likely would you be to enroll your child in a registry if it was only offered through the Internet and you needed to create a user name and password?

◯ Very Unlikely
◯ Somewhat Unlikely
◯ Somewhat Likely
◯ Very Likely

How likely would you be to enroll your child in a registry if it was only offered through email?

◯ Very Unlikely
◯ Somewhat Unlikely
◯ Somewhat Likely
◯ Very Likely

How likely would you be to enroll your child in a registry if it was advertised on a social media site (i.e. Facebook, Instagram, Twitter)?

◯ Very Unlikely
◯ Somewhat Unlikely
◯ Somewhat Likely
◯ Very Likely

How likely would you be to enroll your child in a registry if it was offered by a research nurse in clinic?

◯ Very Unlikely
◯ Somewhat Unlikely
◯ Somewhat Likely
◯ Very Likely

How likely would you be to enroll your child in a registry if it was offered by a research nurse via a Telehealth appointment?

◯ Very Unlikely
◯ Somewhat Unlikely
◯ Somewhat Likely
◯ Very Likely

How likely would you be to enroll your child in a registry if it was offered by your child's primary physician or specialist?

◯ Very Unlikely
◯ Somewhat Unlikely
◯ Somewhat Likely
◯ Very Likely

How willing would you be to provide your child's demographic information (for example age, race, zip code) to a potential participants registry so that you could be matched with appropriate studies?

◯ Very Unlikely
◯ Somewhat Unlikely
◯ Somewhat Likely
◯ Very Likely

How willing would you be to provide your child's medical information (such as your previous diagnoses and current medications) to a potential participants registry so that you could be matched with appropriate studies?	◯ Very Unlikely<br>◯ Somewhat Unlikely<br>◯ Somewhat Likely<br>◯ Very Likely
How willing would you be to allow researchers to link your child's electronic health records to a potential participants registry so that they could be matched with appropriate studies based on their personal medical information?	◯ Very Unlikely<br>◯ Somewhat Unlikely<br>◯ Somewhat Likely<br>◯ Very Likely
How willing would you be to provide lifestyle information (about diet and exercise) about your child to a potential participants registry so that you could be matched with appropriate studies?	◯ Very Unlikely<br>◯ Somewhat Unlikely<br>◯ Somewhat Likely<br>◯ Very Likely
How would it affect your decision whether to enroll your child in a potential participants registry if registrants received new information about health, based on research happening at Children's/UNMC?	◯ Very Unlikely<br>◯ Somewhat Unlikely<br>◯ Somewhat Likely<br>◯ Very Likely
If you enrolled your child in a potential participants registry, how concerned would you be about inappropriate sharing of your information with other doctors and health care professionals at Children's/UNMC?	◯ Very Unlikely<br>◯ Somewhat Unlikely<br>◯ Somewhat Likely<br>◯ Very Likely
If you enrolled your child in a potential participants registry, how concerned would you be about inappropriate sharing of your child's information with other doctors and health care professionals outside of Children's/UNMC?	◯ Very Unlikely<br>◯ Somewhat Unlikely<br>◯ Somewhat Likely<br>◯ Very Likely
If you enrolled your child in a potential participants registry, how concerned would you be about inappropriate sharing of your child's information with insurance companies?	◯ Very Unlikely<br>◯ Somewhat Unlikely<br>◯ Somewhat Likely<br>◯ Very Likely
If you enrolled your child in a potential participants registry, how concerned would you be about inappropriate sharing of your child's information with for-profit companies like the pharmaceutical industry?	◯ Very Unlikely<br>◯ Somewhat Unlikely<br>◯ Somewhat Likely<br>◯ Very Likely
If you enrolled your child in a potential participants registry, how concerned would you be about receiving unwanted telephone communication?	◯ Very Unlikely<br>◯ Somewhat Unlikely<br>◯ Somewhat Likely<br>◯ Very Likely
If you enrolled your child in a potential participants registry, how concerned would you be about receiving unwanted mail communication (letters, flyers, and brochures)?	◯ Very Unlikely<br>◯ Somewhat Unlikely<br>◯ Somewhat Likely<br>◯ Very Likely
If you enrolled your child in a potential participants registry, how concerned would you be about receiving unwanted electronic communication (email)?	◯ Very Unlikely<br>◯ Somewhat Unlikely<br>◯ Somewhat Likely<br>◯ Very Likely

If you enrolled your child in a potential participants registry so that your child could be matched with appropriate studies, what is the longest amount of time you would be willing to spend providing information about your child (at the time of registration)?	◯ 5 minutes<br>◯ 15 minutes<br>◯ 30 minutes<br>◯ 60 minutes<br>◯ 90 minutes
If you enrolled your child in a potential participants registry so that your child could be matched with appropriate studies, which answer below best represents the number of times that would be the most you would be willing to be contacted?	◯ None (I would not want to be contacted at all)<br>◯ Once very two years<br>◯ Once a year<br>◯ 2-3 times a year<br>◯ Monthly<br>◯ No limit (I am happy to be contacted about as many studies as possible)
If your child was enrolled in a potential participants registry, which of the following would be an "acceptable" method to let you know about studies for which they might be eligible? (Mark all that apply)	☐ Telephone Call<br>☐ Text<br>☐ E-mail<br>☐ Mailed letter/flyer
If your child enrolled in a potential participants registry, which of the following would be your "preferred" method to let you know about studies for which your child might be eligible?	☐ Telephone Call<br>☐ Text<br>☐ E-mail<br>☐ Mailed letter/flyer
